# Dynamic Behavior of Bound Interlayer Excitons in Interlayer-Doped Cs_3_Bi_2_Br_9_ Vacancy-Ordered Perovskite

**DOI:** 10.1021/acsnano.5c14651

**Published:** 2025-10-16

**Authors:** Kyeongdeuk Moon, Yang Ding, Halyna Okrepka, Rihan Wu, Caitlin N. Ewald, Pushpender Yadav, Anupam Biswas, Elad Harel, Masaru Kuno, Seokhyoung Kim

**Affiliations:** † Department of Chemistry, 3078 Michigan State University, East Lansing, Michigan 48824, United States; ‡ Department of Chemistry and Biochemistry, 6111 University of Notre Dame, Notre Dame, Indiana 46556, United States

**Keywords:** vacancy-ordered perovskite, exciton, bound interlayer exciton, interlayer doping, single-particle spectroscopy

## Abstract

Interlayer doping of the vacancy-ordered 2D perovskite Cs_3_Bi_2_Br_9_ (CBB) enables the formation of bound interlayer excitons (BIEs), a unique charge-transfer excited state within the layered solid. BIEs previously reported with silver (Ag^+^) as an interlayer dopant exhibited bright broadband photoluminescence (PL) with prolonged lifetime at room temperature, offering potential applications in efficient white light emission, photocatalysis, and optoelectronics. However, the dynamic behavior of radiation and excited carriers remains poorly understood due to the limitations of ensemble spectroscopic measurements. Here, we investigate the temperature-dependent dynamics of Ag-doped Cs_3_Bi_2_Br_9_ (Ag-CBB) using single-particle time-resolved PL spectroscopy and ultrafast transient absorption imaging. Single-particle PL measurements reveal three distinct emission regimes across temperature: (i) BIE-dominant emission at high temperatures, (ii) a mixture of radiation from BIEs and self-trapped excitons (STEs) at intermediate temperatures, and (iii) STE-dominant emission below 100 K. Rapid transient absorption mapping using Parallel Rapid Imaging with Spectroscopic Mapping (PRISM) reveals subpicosecond STE formation in pristine CBB and long-lived photoinduced absorption by BIEs, consistent with electron–hole separation and suppressed STE transfer. The spatial uniformity of these signals confirms homogeneous Ag doping across single crystals. These findings highlight the role of Ag interlayer dopants in governing the BIE dynamics.

## Graphical abstract

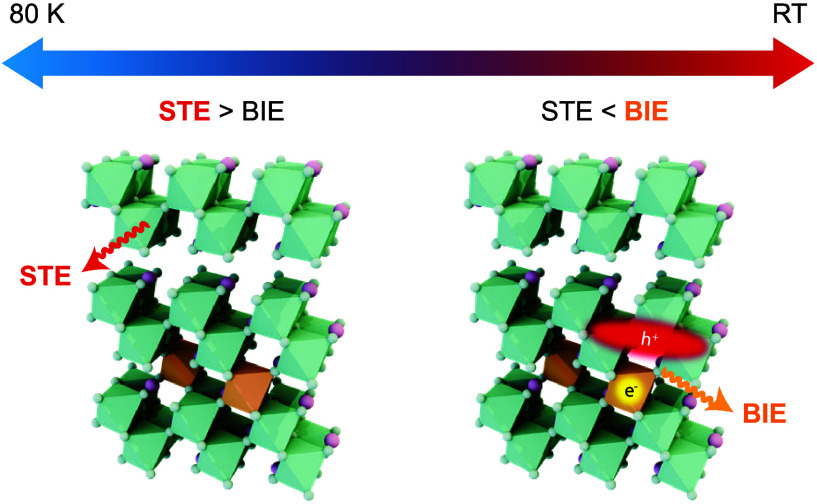

## Introduction

Materials discovery research on halide perovskites has grown rapidly due to their remarkable optoelectronic properties and broad applications in solar energy conversion, light-emitting diodes, photodetection, and X-ray scintillation.^1–6^ Among the various strategies, dimensional engineering has proven to be one of the most effective approaches for tuning excitonic and radiative behaviors.^7–10^ Reducing the dimensionality of halide perovskites from three-dimensional (3D) frameworks to two-dimensional (2D) layered structures strongly confines charge carriers with reduced dielectric screening, leading to enhanced exciton binding energies and improved radiative recombination rates, both of which are beneficial for efficient light emission.^11,12^

2D vacancy-ordered halide perovskites with the A_3_B_2_X_9_ formula naturally exhibit layered crystal structures. Among them, lead-free Cs_3_Bi_2_Br_9_ (CBB) and Cs_3_Sb_2_Br_9_ have emerged as promising candidates that combine the advantages of reduced dimensionality and environmental sustainability.^13–16^ This class of perovskites also exhibits a significantly greater stability in a wide range of common solvents, such as protic alcohols, in which lead halide perovskites degrade rapidly.^17^ Such stability enables their compatibility with well-established lithographic processes for device integration. However, in their pristine forms, their optoelectronic performance hinders practical applications. For instance, CBB exhibits weak photoluminescence (PL) at room temperature, originating from the presence of an indirect bandgap slightly below the direct bandgap, making radiative recombination inefficient.^18,19^ Additionally, CBB shows strong electron–phonon scattering, leading to significant nonradiative dissipation of absorbed energy at room temperature.^20^ As a result, despite its structural advantages, pristine CBB suffers from low PL quantum yield (QY) under ambient conditions.^21,22^ While recent advances in the synthesis of colloidal quantum dots (QDs) and various surface engineering strategies have markedly enhanced emission strengths, the reported PLQY yields still remain relatively low^23,24^ compared to near-unity PLQY of lead-based perovskite QDs.^25^

Recently, we discovered that the neutral vacancies present between neighboring CBB layers can be harnessed as a crystalline doping site, yielding an interlayer doping configuration.^18^ Figure 1A depicts the crystal structure of CBB and the interlayer doping site located in the middle of two adjacent unit cells. Using controlled chemical vapor deposition (CVD) growth with silver (Ag^+^) as an interlayer dopant, we reported that the Ag atom occupies an interlayer vacancy in CBB, and its +1 charge is balanced by the loss of Cs^+^ ions. We demonstrated the emergence of a new strong PL channel in Ag-doped CBB (Ag-CBB) at an approximate 1.8% doping level, which transformed CBB from a nearly dark emitter into a strong broadband emitter at room temperature. The new emission channel is attributed to the formation of bound interlayer exciton (BIE), a unique charge-transfer exciton state that displays the characteristics of bound excitons (BEs)^26,27^ and interlayer excitons (IEs)^28–30^ at the same time. Briefly, with Ag^+^ acting as an electron trap, photoexcited electrons are instantly trapped into the vacancy region, leading to an interlayer separation of electrons and holes. This separation reduces wave function overlap and thereby extends the lifetime of these excited carriers. Additionally, due to the localized nature of Ag^+^, the separated electron–hole pairs are spatially bound to local dopants, resulting in immobile, bound exciton species. These bound and interlayer characters collectively develop a strong PL intensity with a radiative decay time over an order of magnitude greater than that of free excitons (FEs) in the same parent material.

**Figure 1:**
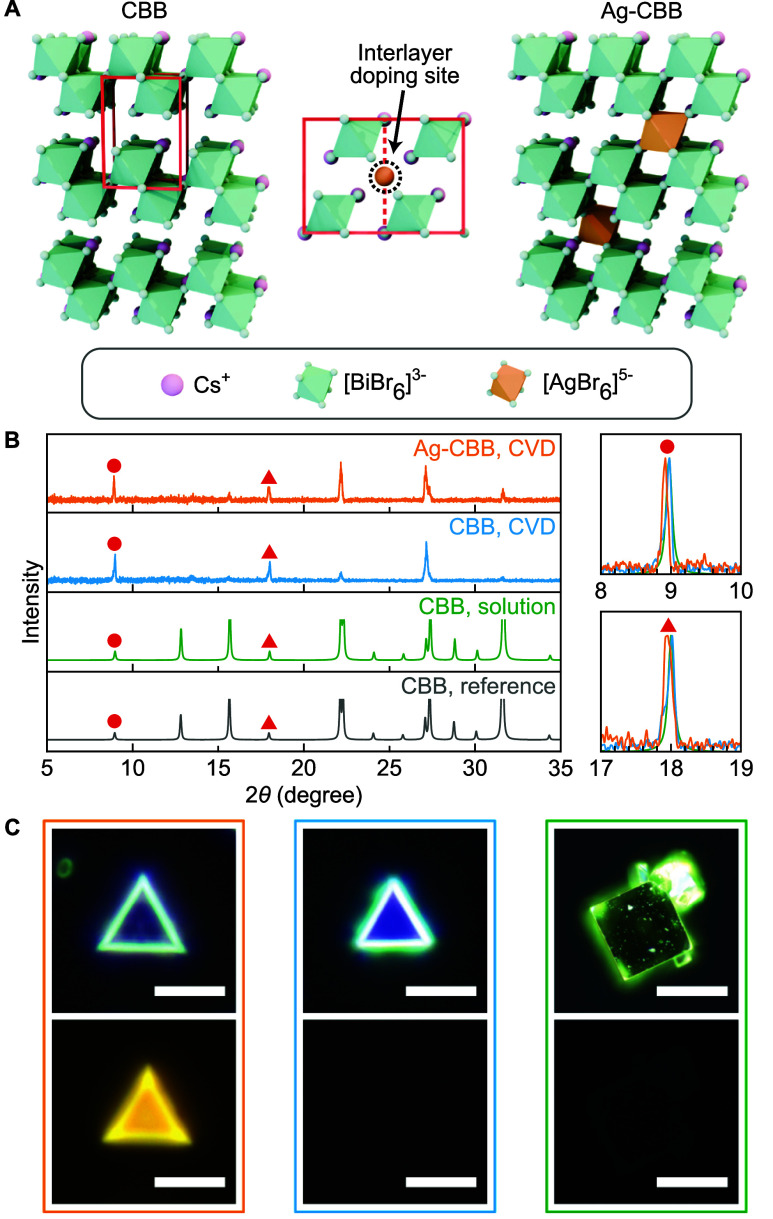
Structural analysis. (A) Crystal structures of CBB (left), interlayer doping site (middle), and Ag-CBB (right). The red box in CBB structure indicates the unit cell. (B) Experimental and simulated XRD patterns. The insets on the right show magnified views around two fundamental peaks. (C) Optical DF (top) and PL (bottom) images of CVD-grown Ag-CBB (left), CVD-grown CBB (middle), and solution-synthesized CBB (right); scale bars are 5 μm for CVD-grown Ag-CBB and CBB, and 100 μm for solution-synthesized CBB.

Despite these intriguing demonstrations, the dynamic behavior of BIEs has not yet been fully understood. Previous PL measurements on a large ensemble of micron-size Ag-CBB single crystals showed a single strong BIE PL band centered at 600 nm, with little change in spectral features when the temperature was lowered from room temperature to 80 K. In contrast, pristine CBB is known to exhibit blue FE emission at about 470 nm near room temperature and to develop a strong near-infrared (NIR) STE emission band around 720–770 nm below 200 K.^19^ Thus, a competitive coexistence of emission from all three species—FE, BIE, and STE—would be expected. However, this effect was not captured in the previous ensemble PL measurements, leaving unresolved questions about the fundamental behavior of excited carriers at early times. Achieving correct and accurate understanding of the carrier movement is crucial for the well-informed design of devices and interfaces for further property engineering.

In this study, using time-resolved single-particle spectroscopy, we investigate the temperature-dependent radiative recombination dynamics of Ag-CBB and demonstrate emission transitions among the participating exciton species. A single-particle measurement is key to eliminating interparticle variations in the extrinsic effects and revealing the complex interplay among FEs, STEs, and BIEs—an effect unresolved in ensemble measurements. We identified an emission crossover from a BIE-dominant high-temperature regime to STE-dominant low-temperature regime in Ag-CBB. FE emission was rarely observed in Ag-CBB single crystals at any temperature, supporting rapid trapping of photoelectrons by Ag dopants. The exact temperatures at which the STE onset or BIE cutoff occurs varied, indicating that the PL transition depends on external factors such as particle size and doping concentration at a given excitation power—reflecting the extrinsic nature of doping.

Throughout this temperature range, we observed a dramatic change in the radiative decay time. Due to reduced phonon activity with decreasing temperature, the BIE decay time increases by nearly 500% from 48 ns at room temperature reaching up to 250 ns at 160 K, which is over 2 orders of magnitude longer than that of FE in pristine CBB. Upon further cooling, decay time reverts back to subnanosecond, as STE takes over the dominant emission mechanism.^19^

Through ultrafast transient absorption (TA) imaging, we reveal that the rapid quenching of photocarriers into the BIEs prevents the formation of STEs, resulting in a much more prolonged transient response compared to undoped pristine CBB. This effect occurs homogeneously across individual single particles, confirming the uniform distribution of dopants and the absence of other localized inhomogeneities. Our findings provide detailed insight into dopant-mediated exciton dynamics in interlayer-doped vacancy-ordered perovskites for advancing their potential for light-emitting applications.

## Result

### Structural Characterization

CBB and Ag-CBB microcrystals were synthesized using a home-built CVD system with CsBr, BiBr_3_, and AgBr as source precursors (Figures S1 and S2). For a fair comparison with existing literature, CBB was also prepared using a standard solution-based method.^19^ The powder X-ray diffraction (XRD) patterns in Figure 1B confirm that CBB synthesized by both methods is structurally identical. In CVD-grown CBB, only (000*l*) reflections are predominantly observed due to a highly oriented layer-by-layer growth behavior of CVD synthesis. Nonetheless, both methods yield XRD patterns that match well with the calculated reference pattern. Ag-CBB displays an XRD pattern near identical to that of CVD-grown CBB. A detailed comparison of the (0001) and (0002) peaks denoted with circles and triangles, respectively, is presented on the right side of Figure 1B. As reported in our initial study, both the (0001) and (0002) peaks are slightly shifted toward lower angles in Ag-CBB, attributed to expanded interlayer spacing resulting from the insertion of Ag dopants between CBB layers.^18^

Optical dark-field (DF) and PL images of all three samples are shown in Figures 1C and S3. CVD-grown crystals exhibit triangular shapes due to the 3-fold symmetry of the hexagonal *ab* plane. On the other hand, solution-grown CBB crystals exhibit cubic morphology with significantly larger sizes, attributed to more isotropic growth in solution, which favors the formation of equi-dimensional crystal shapes. For PL characteristics, Ag-CBB exhibits bright yellow emission around 600 nm at room temperature when excited with a 380 nm LED source. This strong emission arises from the interlayer Ag dopants and is absent in undoped CBB crystals, both CVD- and solution-synthesized, consistent with the near-dark PL of CBB reported in the literature.^31–33^

### Temperature-Dependent PL

Temperature-dependent PL spectra of CBB and Ag-CBB were acquired from 240 to 80 K, as shown in Figure 2. For undoped CBB, solution-grown samples were used because the PL from CVD-grown CBB is often too weak to be experimentally detected, owing to their small sizes and intrinsically low PLQY. In such cases, the use of much larger solution-grown CBB single crystals enables a direct and practical comparison with previously reported literature.^6,19^ At 240 K, CBB exhibits two emission bands: a strong FE emission at 475 nm, corresponding to the 2.6 eV direct bandgap transition, and STE emission beginning to emerge at 725 nm.^31^ The PL image at this temperature shows a very weak blue color (Figure 2A, left). As the temperature decreases, the FE peak rapidly diminishes while the STE peak grows (Figure 2C). This transition is attributed to the greater stability of the polarized lattices surrounding STEs,^19,34^ which also gives rise to the observed STE PL red-shift at lower temperatures (Figure 2A, right). Across the temperature range, no emission peak is observed within the spectral window corresponding to BIE emission (yellow-shaded region in Figure 2C).

**Figure 2:**
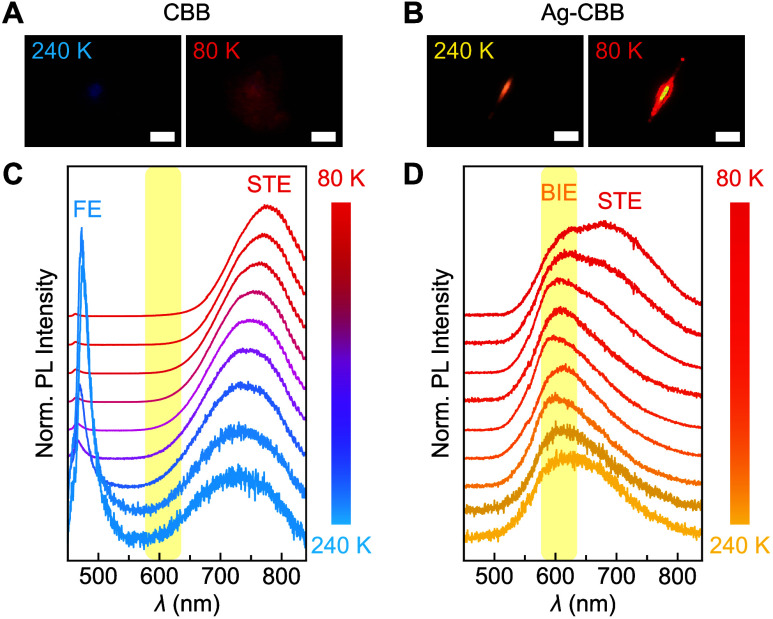
Temperature-dependent PL. (A, B) PL images of solution-grown CBB (A) and Ag-CBB (B) at 240 K (left) and 80 K (right); all scale bars, 5 μm. (C, D) PL of CBB (C) and Ag-CBB (D) acquired from 80 to 240 K with 20 K increment. Each curve is vertically offset for clarity. Yellow shades represent the spectral band of BIE emission.

Ag-CBB, on the other hand, exhibits a single broad PL peak centered at 600 nm at 240 K, consistent with radiative recombination of BIEs. At this temperature, neither FE nor STE emission peaks are observed, and the PL image shows a yellow color (Figure 2B, left). As the temperature is lowered, an STE peak begins to emerge on the shoulder of the BIE peak, showing a distinct apex around 100 K and becoming distinctly visible at 680 nm (Figure 2D). At 80 K, the PL image shows a red color (Figure 2B, right), similar to that observed for CBB at the same temperature.

Two clear differences are observed in the PL behavior of Ag-CBB compared to CBB. First, the FE PL peak is not detected in Ag-CBB at any temperature. This complete quenching of FEs is attributed to the rapid trapping of photoelectrons by the Ag trap state located near the conduction band minimum.^18^ This FE-to-BIE transfer process dominates at high temperatures, leading to the exclusive observation of the 600 nm BIE emission. The BIE state has previously been found to exhibit a 2.14 eV transition energy, which matches the 600 nm PL center wavelength. Second, the center wavelength of STE PL in Ag-CBB (680 nm) notably differs from that of CBB (775 nm). This variation in the Stokes shifts of the STE emission is attributed to local differences in the lattice polarization, arising from the presence or absence of interlayer Ag dopants.^19^

Also noteworthy is that we observed an extrinsic characteristic in BIE emission intensity across Ag-CBB single particles. We found that, when measured at 120 K, BIE emission exhibited a pronounced sublinear saturation behavior as a function of excitation power (Figure S4), whereas FE emission showed a linear dependence. STE emission also exhibited sublinearity, though to a lesser extent than BIE.^35^ This corroborates the limited availability of dopants and a faster saturation of BIE.^36^ Furthermore, while the 80 K emission curve in Figure 2D shows the presence of both BIE and STE, we often observed Ag-CBB particles dominated by STEs under temperatures and excitation conditions with BIE emission suppressed at relatively higher temperatures, as will be discussed in the next section (Figure 3D). We attribute this interparticle variation to extrinsic effects, including differences in particle size and dopant number. Therefore, when PL from a large ensemble of Ag-CBB particles is measured under global illumination, the result contains substantial ensemble inhomogeneity, which explains the absence of spectral changes in temperature-dependent ensemble PL in the previous study.^18^

**Figure 3:**
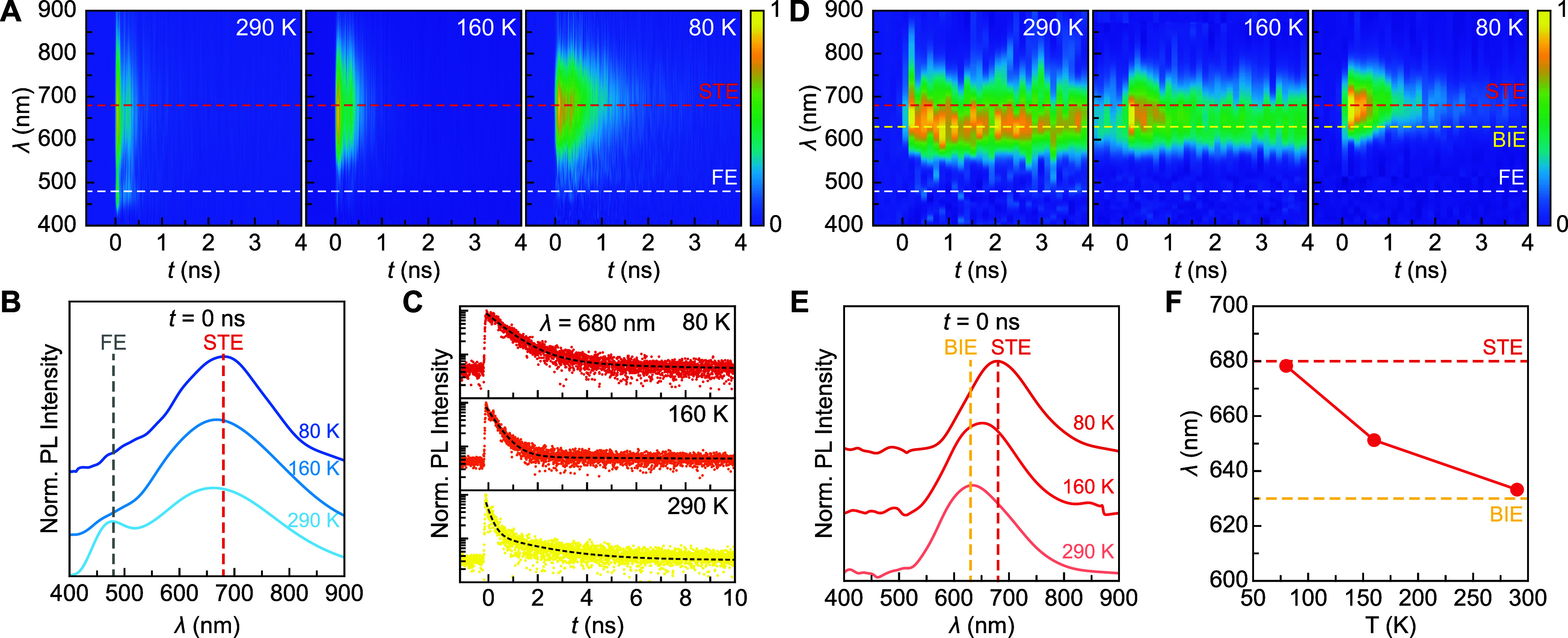
Temperature-dependent TRPL. (A) PL heatmaps of CBB as functions of *t* (horizontal axis) and λ (vertical axis) acquired at 290, 160, and 80 K. (B) PL spectra at *t* = 0 at three temperatures from panel (A). Dashed lines represent FE (gray, 480 nm) and STE (red, 680 nm) emission center wavelengths. The curves are vertically offset for clarity. (C) TRPL decay curves of CBB at 680 nm from panel (A) with biexponential fits overlaid (black dashes). (D) PL heatmaps of Ag-CBB as functions of *t* (horizontal axis) and λ (vertical axis) acquired at 290, 160, and 80 K. (E) PL spectra at *t* = 0 at three temperatures from panel (D). Dashed lines represent BIE (yellow, 630 nm) and STE (red, 680 nm) emission center wavelengths. The curves are vertically offset for clarity. (F) PL peak maxima of Ag-CBB at three temperatures (red circles). Dashed guidelines represent BIE and STE emission wavelengths.

### Temperature-Dependent TRPL

To further investigate the complex interplay of FE, STE, and BIE, time-resolved PL spectroscopy (TRPL) was performed on CBB and Ag-CBB single particles at three temperatures: 290, 160, and 80 K. The TRPL of a pristine CBB single particle is shown in Figure 3A. At 290 K, it reveals two distinct peaks at 480 and 680 nm, corresponding to FE and STE emission, respectively, both of which decay rapidly within 1 ns. A quantitative fit to the FE emission decay (480 nm) shows a single-exponential profile with a 0.32 ns lifetime (Figure S5). The FE peak becomes indistinguishable as the temperature decreases to 160 and 80 K (Figure 3B), therefore its decay dynamics are not further analyzed. On the other hand, the position of STE PL remains nearly unchanged at 680 nm at all temperatures (Figure 3B). The temporal STE PL profiles show a decay time (τ) that increases as the temperature decreases. This behavior is characteristic of STEs, as reduced thermal energy suppresses nonradiative relaxation pathways, leading to longer τ at lower temperatures.^37^ The τ_*i*_ and their relative amplitudes (*a*_*i*_) obtained from fitting to a biexponential function are summarized in Table 1.

**Table 1:** STE PL Decay times and Amplitudes at 680 nm in CBB

*T* (K)	τ_1_ (ns)	*a*_1_ (%)	τ_2_ (ns)	*a*_2_ (%)	τ_avg_ (ns)
290	0.21	84.8	1.97	15.2	0.48
160	0.46	98.6	17.81	1.4	0.70
80	0.85	95.4	4.26	4.6	1.01

Ag-CBB single particle exhibits two distinct PL behaviors. First, its emission at 290 K is centered at 630 nm (yellow dashed line in Figure 3D), which is about 50 nm blue-shifted from the STE peak observed in CBB (red dashed line). The temporal decay profile of this emission shows a much slower depopulation rate, with the decay tail extending beyond the time range shown in Figure 3D. Full decay traces of Ag-CBB plotted over a longer time range are presented and discussed in Figure 4. Second, as the temperature is lowered, the decay rate rapidly shortens, and the emission envelope gradually red-shifts to 680 nm, which we assign to STE emission. At 80 K, the TRPL heatmap of Ag-CBB (rightmost in Figure 3D) restores most features of the TRPL heatmap of CBB (rightmost in Figure 3A), indicating the presence of comparable STE dynamics.

**Figure 4:**
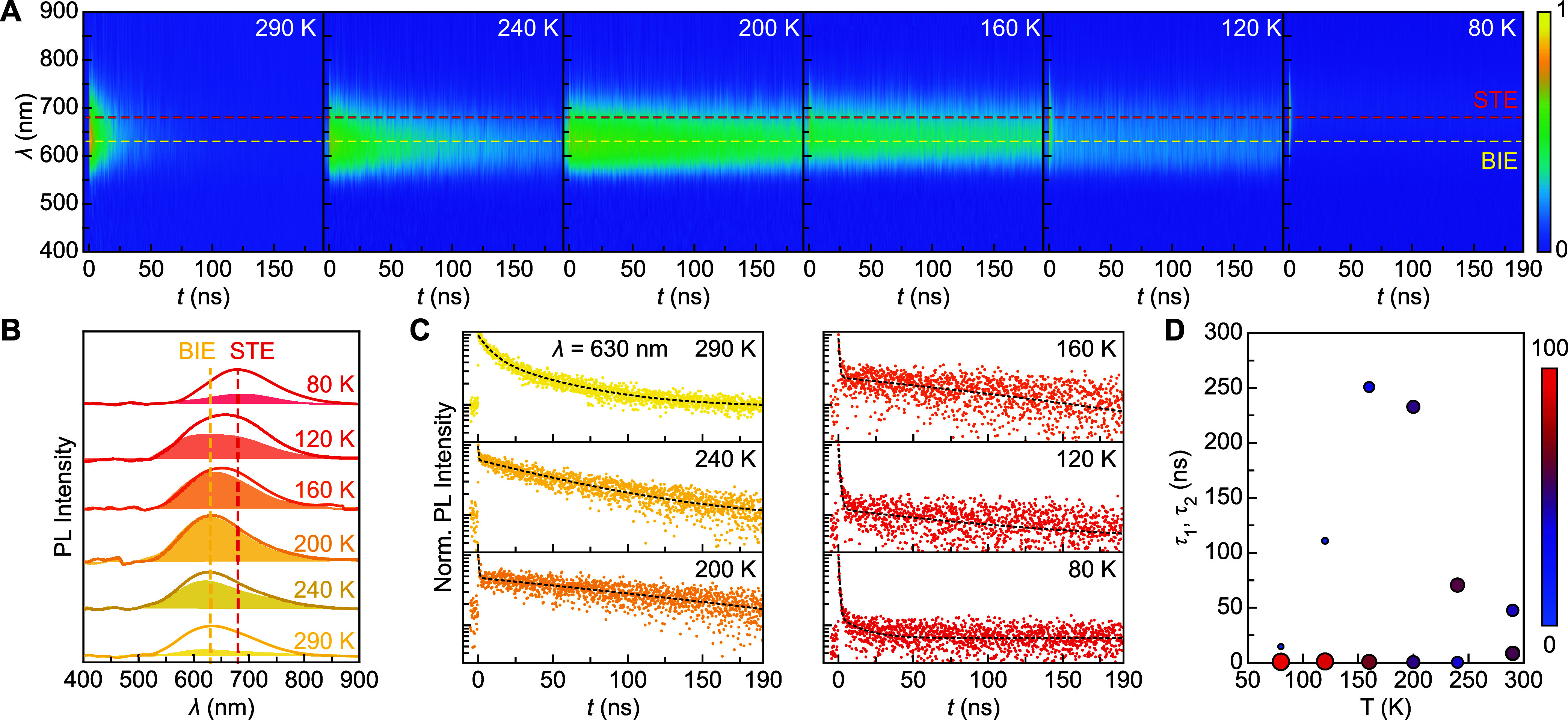
Temperature-dependent TRPL of Ag-CBB. (A) TRPL heatmaps of Ag-CBB from 290 to 80 K with 40 K intervals, plotted up to 190 ns. (B) PL spectra of at *t* = 0 (solid line) and *t* = 50 ns (shaded area). All shades are magnified by 10 times for clarity. (C) Decay traces at 630 nm with fits to biexponential functions. (D) Extracted decay times for different temperatures. The size and color of the circles represent relative amplitudes of τ_1_ and τ_2_.

The broad line widths of both BIE and STE PL present a challenge for independently resolving the detailed photophysical behavior of these two excitonic species. To facilitate a simple comparison, we first examine the PL curves at *t* = 0 for all three temperatures in Figure 3E. Unlike the STE emission in CBB that shows a negligible spectral shift with temperature (Figure 3B), the PL envelope of Ag-CBB shows a subtle yet monotonic shift from 630 nm at 290 K to 680 nm at 80 K. When the peak maxima are plotted along with known wavelengths of BIEs and STEs (Figure 3F), a clearer picture emerges: BIE dominates emission at high temperatures, while STEs become the primary mechanism at low temperatures. The decay times observed at 290 and 80 K further support this exciton transition. At 290 K, the 630 nm emission exhibits a long decay time beyond 4 ns, which differs significantly from CBB’s emission (680 nm, τ < 1 ns), supporting its assignment to BIE that is absent in CBB.

Overall, competition between BIE and STE emissions in Ag-CBB is clearly evident. To quantitatively examine the relationship between these two excitonic species, TRPL measurements were conducted over an extended time window (190 ns) and are presented in Figure 4A across six temperatures. First, we examine the two extreme temperatures, 290 and 80 K. In this case, the previously described behavior—the PL transition from BIE (630 nm) to STE (680 nm)—is observed.

However, at intermediate temperatures, a much more dynamic change is observed. To initially examine the temporal evolution of spectral shapes, PL curves at *t* = 0 and 50 ns are plotted in Figure 4B, revealing three distinct regimes. At temperatures between 290 and 200 K, the PL peak position remains consistently at 630 nm with no observable spectral peak shift over time. This region is identified as a BIE-dominant high-temperature regime.

At 160 and 120 K, the PL curves exhibit a slight spectral shift between *t* = 0 and 50 ns and overall broader line widths, which we attribute to the spectral overlap of BIE and STE emissions. At 160 K, a noticeable blue-shift is observed at *t* = 50 ns. Because STEs typically exhibit shorter decay times than BIEs, we interpret this shift as an increased contribution of BIE emission at later times due to faster STE decay. This behavior is more pronounced at 120 K, where the *t* = 50 ns PL begins to show a bifurcation into two PL shoulders. Therefore, this second range is defined as an intermediate regime, where BIEs and STEs coexist and dynamically compete.

At the lowest temperature of 80 K, both PL curves at *t* = 0 and 50 ns shift entirely to the STE band around 680 nm. This represents a third STE-dominant low-temperature regime where BIE emission is absent.

With these three regimes identified, their temporal decay profiles are examined. Figure 4C displays decay traces at the BIE-emitting 630 nm, fitted with a biexponential function. Due to the broad line widths and spectral overlap, decay traces at the STE-emitting 680 nm show similar results (see Figure S6). The extracted τ_*i*_ and *a*_*i*_ are summarized in Table 2 and visually presented in Figure 4D where the vertical position and sizes of circles represent the τ_*i*_ and a_*i*_, respectively. In the BIE-dominant regime, the slower component τ_2_ more accurately represents the intrinsic dynamic behavior of BIEs, while the faster component τ_1_ likely arises from multiple unwanted processes such as nonradiative processes and energy back transfer from Ag to the host lattice. Additionally, to our surprise, the BIE τ_2_ monotonically increases from 48 ns to over 200 ns, eventually reaching a maximum value of 250 ns at 160 K. For comparison, the FE emission τ in undoped CBB is approximately ∼1 ns, indicating a 250-fold increase in the exciton lifetime.^18^ Below 120 K, however, the BIE τ_2_ rapidly decreases and becomes negligible at 80 K. Instead, the faster component τ_1_ converges to ∼1 ns, consistent with the typical STE emission τ of host CBB.

**Table 2:** TRPL Decay Parameters of Ag-CBB at 630 nm

*T* (K)	τ_1_ (ns)	*a*_1_ (%)	τ_2_ (ns)	*a*_2_ (%)	τ_*avg*_ (ns)
290	8.48	57	47.58	43	25.59
240	0.32	40	70.57	60	42.47
200	0.51	51	228.56	49	112.25
160	0.74	67	250.99	33	83.32
120	1.11	90	110.78	10	12.08
80	0.76	93	14.52	7	1.72

### Ultrafast Transient Absorption Imaging

To further investigate the excited-state dynamics of CBB and Ag-CBB, ultrafast TA measurements were performed using Parallel Rapid Imaging with Spectroscopic Mapping (PRISM).^38^ Unlike conventional broadband TA techniques that provide ensemble-averaged spectral dynamics and thus limited spatial information, PRISM provides simultaneous spatial and temporal resolution to capture local variations in the early excited-state behavior by generating two-dimensional (2D) spatial maps of TA signals. A 750 nm pump was used to drive two-photon excitation, enabling bulk-sensitive generation of free carriers while minimizing linear absorption and scattering. A 650 nm probe was selected to track photoinduced absorption (PIA) of excited carriers.^39^ The TA traces presented in Figure 5B,D are taken from the red-boxed regions of the respective samples, with additional curves from gray regions provided in Figure S7.

**Figure 5:**
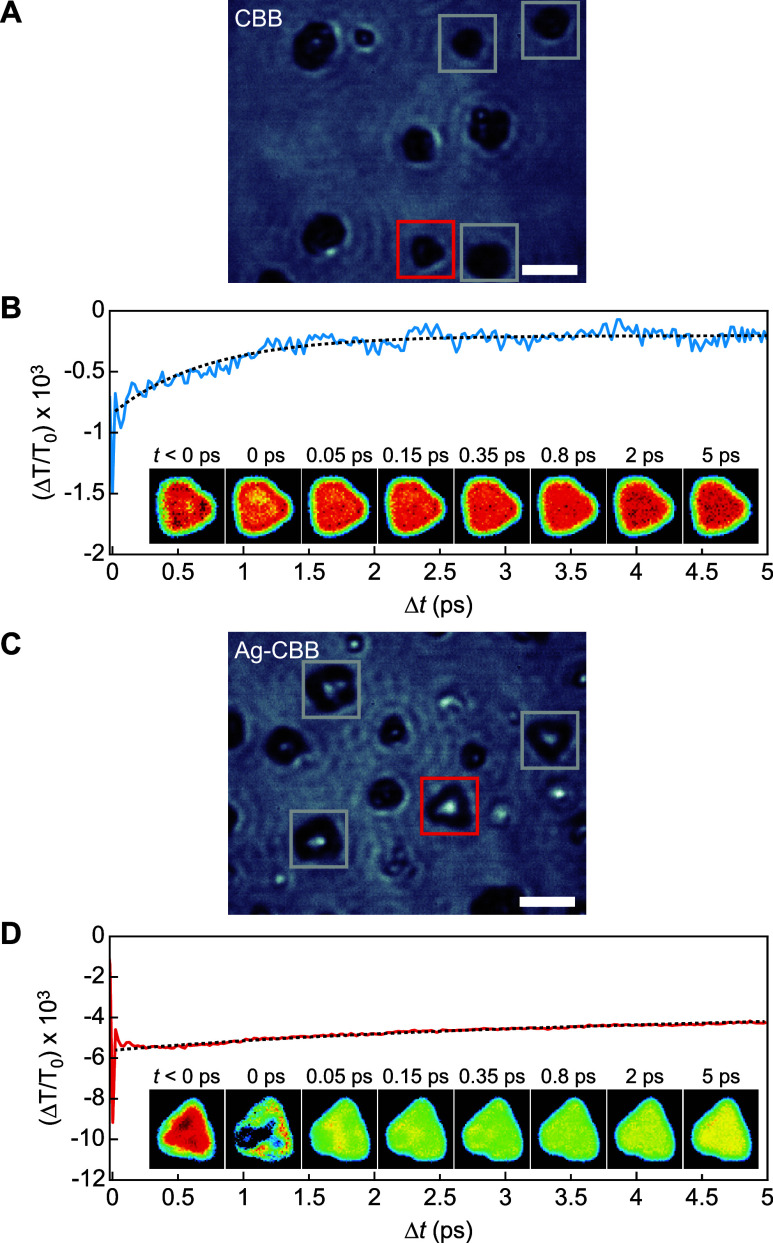
Ultrafast TA imaging of Ag-CBB and CBB. (A, C) Optical images of CBB (A) and Ag-CBB (C); scale bars, 20 μm. (B, D) Ultrafast TA kinetics from single crystal within the red box for CBB (B) and Ag-CBB (D), showing the normalized differential transmittance as a function of delay time. Inset images show percent transmittance change at various delay times.

Based on the kinetic traces, a weak TA signal is observed for a pristine CBB (Figure 5A), with a PIA decay time of 0.7 ps under single-exponential fitting. This rapid decay indicates that free carriers initially absorb the 650 nm probe beam upon excitation but are quickly lost to other states that no longer absorb. Two possible processes are considered as the origin of this rapid decay: defect-mediated nonradiative recombination and STE formation. The defect-mediated pathway is ruled out because it would result in a complete loss of carriers, which cannot explain the FE PL observed at later times. The observation of STE PL at room temperature (Figure 3A) therefore supports the STE formation as the more plausible explanation, which also aligns with previous reports of picosecond-scale STE formation kinetics.^40,41^

In Ag-CBB, in contrast, the TA trace could only be accurately represented when fitted with a biexponential profile (Figure 5D). The fast decay component has a time constant of 2.0 ps, qualitatively comparable with the 0.7 ps decay of pristine CBB. This process accounts for 14.8% of the total amplitude, corresponding to free carriers forming STEs. The slow component, however, with a dominant amplitude of 85.2%, exhibits a decay time far exceeding the 10 ps delay range and could not be accurately determined. This ≫10 ps process indicates that free carriers absorbing the 650 nm probe light persist for much longer without forming STEs. This process is not intuitively understood because photocarriers would typically follow the pathway to STE formation and vanish rapidly. The presence of a long-lasting PIA signal instead suggests that STE formation is strongly suppressed by another mechanism enabled by Ag dopants. According to the previous study, interlayer Ag dopants act as electron traps, leaving free holes in the CBB layers.^18^ This offers two possible explanations. First, once photoelectrons are trapped into the spacer region by the dopants, the remaining holes can no longer form STEs on their own. Second, the long-lived TA signal suggests that the PIA arises from an intravalence band transition of these photoholes. Therefore, the electron–hole separation prevents excited holes from transferring to STE states, enabling continued PIA of the probe light for extended times. As shown in the insets of Figure 5B,D, the TA signal is barely noticeable in the pristine CBB single crystal but is observed uniformly across the entire Ag-CBB single particle, with no sign of localized domains. This spatial uniformity confirms homogeneous carrier dynamics in Ag-CBB.

## Conclusion

In conclusion, we investigated the temperature-dependent exciton dynamics in interlayer-doped Ag-CBB single crystals using steady-state and TRPL spectroscopy. Our results reveal clear competition between BIEs and STEs across a broad temperature range. At room temperature, BIE emission dominates, with no detectable STE or FE emission. The BIEs exhibit a long decay lifetime of about 48 ns which is over 40 times longer than that of FEs or STEs in undoped CBB, and this lifetime increases up to 250 ns as the temperature decreases. STE emission begins to dominate over BIE emission as exciton localization becomes more pronounced. Ultrafast TA imaging with PRISM revealed significantly long-lived PIA, representing trapped electrons and suppressed STE formation. The spatial uniformity of this decay across the single particle indicates spatially homogeneous optoelectronic activity. These findings advance our understanding of exciton behavior in interlayer-doped vacancy-ordered perovskites and highlight that dopant-induced modifications can tune exciton pathways, offering a promising approach for developing optoelectronic devices with long-lived tunable emission.

## Methods

### CVD Synthesis of CBB and Ag-CBB

CBB and Ag-CBB were grown on microscope glass slides cut to 1 cm × 1 cm or on SiO_2_ wafers (Nova Electronic Materials; n-type (100) Si with 3000 Å thermal oxide; 1–10 Ω cm) cut to 2 cm × 1 cm in our home-built CVD system. Substrates were first cleaned by sonication in acetone (Sigma-Aldrich; ACS reagent, ≥99.5%) for at least 15 min, rinsed with acetone and isopropanol (Sigma-Aldrich; ACS reagent, ≥99.5%), blow-dried with air, and treated in a UV-Ozone cleaner (Samco UV-1) at 150 °C for 5 min. The quartz tube was baked at 950 °C for 1 h with 100 sccm Ar gas under vacuum, then cooled to room temperature. BiBr_3_ (Sigma-Aldrich; ≥98%), AgBr (Fisher Scientific; 99.5%), and CsBr (Sigma-Aldrich; ≥99.999% trace metals basis) powders were gently ground using a mortar and pestle, then placed in a quartz boat at the center of a single-zone furnace (Fisher Scientific; Lindberg/Blue M Mini-Mite). Clean substrates were placed 11 cm (CBB) and 10.5 cm (Ag-CBB) downstream from the center of the furnace. The experiment was conducted at 510 °C and 280 Torr with a 70 sccm Ar flow. The temperature was increased at a ramp rate of 50 °C/min to the target temperature. The reaction was carried out for 30 min and then cooled naturally to 200 °C before opening the furnace, while pressure and Ar flow were maintained throughout. Nanowires of the Ag-CBB were grown under the same condition as for Ag-CBB, except that the substrate was changed to SiO_2_.

### Solution Synthesis of CBB

CsBr (0.1 M) and BiBr_3_ (0.067 M) were added to 15 mL hydrobromic acid (HBr, ACS reagent, 48%, Sigma-Aldrich) in a glass vial. The mixture was heated in a silicone oil bath at 80 °C overnight. After complete dissolution, the transparent yellow solution was left to evaporate at room temperature overnight, producing yellow crystals. The crystals were separated from the solution and washed with isopropyl alcohol (IPA, ACS reagent, ≥99.5%, Sigma-Aldrich).

### Powder and Single-Crystal X-ray Diffraction

Powder X-ray diffraction was obtained on a Bruker D8 advance diffractometer using Cu Kα radiation (λ = 1.5418 Å) having a LYNXEYE (1D mode) detector over the 2θ range of 5–50° with a step size of 0.02° for 15 min.

Single crystal X-ray diffraction data of solution-phase synthesized CBB crystals were obtained by mounting a yellow cube-shaped crystal with dimensions of 0.10 × 0.07 × 0.07 mm^3^ onto a nylon loop using Paratone oil. Data were collected using an XtaLAB Synergy, Dualflex, HyPix diffractometer equipped with an Oxford Cryosystems 800 low-temperature device, operating at *T* = 299.98 K. Data were measured using ω scans using Cu K_a_ radiation (microfocus sealed X-ray tube, 50 kV, 1 mA).

### Optical Imaging

Optical images were acquired using a Zeiss upright optical microscope. Dark-field (DF) images were acquired using micro-LED light with a dark filter cube (ZEISS). Photoluminescence (PL) images of CVD-grown CBB and Ag-CBB were taken with a filter cube (bandpass filter 450–490 nm for excitation (ZEISS), and long-pass filter 500 nm for emission (Thorlabs)). PL images of solution-grown CBB were taken using a UV LED flashlight with a filter cube 01 (bandpass filter 365/12 for excitation (ZEISS), and long-pass filter 397 nm for emission (ZEISS)).

### Temperature-Dependent PL

Temperature-dependent PL spectra were acquired using a home-built optical setup. The output of a 405 nm continuous wave (CW) excitation laser light (Coherent, OBIS) was focused onto specimens, mounted to a microscope cryostat (Cryo Industries of America), using a long working distance microscope objective (Nikon, NA = 0.45). Excitation intensities ranged from *I*_exc_ = 4.1 W cm^–2^ to 856 kW cm^–2^. Induced emission was collected with the same objective and was passed through a 442 nm long-pass filter (Semrock) before being dispersed with a monochromator (Acton, SP2300, 150 g/mm, 700 nm blaze). An electron-multiplying CCD camera (Andor, iXon Ultra), controlled by purpose-written python software, recorded resulting spectra (100 frames, 1 ms exposure, electron multiplying gain = 20). The microcrystals were imaged by Nikon microscope (Eclipse Ti–U) using a Nikon objective (CFI S Plan Fluor ELWD, 20×, NA = 0.45) with 1x tube lens. A USB-eyepiece camera (AmScope, MD130,1.3 MP) was used to capture images of microcrystals. The brightness and contrast of the captured images of CBB in Figure 2A were adjusted to reflect the seen colors.

### Temperature-Dependent TRPL

TRPL spectroscopy and time-correlated single-photon counting (TCSPC) measurements were conducted using an interferometer (Nieros, Gemini.) coupled to a single-photon counting avalanche photodiode (MPD) and a time-correlated single-photon counting module (PicoQuant, PicoHarp 300). A pulsed 405 nm diode laser (PicoQuant, LDH–P-C-405), controlled by an external laser driver (PicoQuant, PDL-800-B), was used to excite the samples. The laser operated at a repetition rate of 10 MHz with pulse widths of approximately 70 ps. Pulsed excitation beams were guided into an inverted microscope (Nikon Eclipse Ti2). A 405 nm long-pass beam splitter inside the microscope directed the excitation light into the objective. A long working distance microscope objective (Nikon CF Plan 50×, NA = 0.45, Epi SLWD) focused the laser light onto the specimen inside a cryostat (Lake Shore Cryotronics, ST-500) equipped with a temperature controller (Lake Shore Model 335). The resulting emission was collected with the same objective, filtered with a 442 nm long-pass filter (Semrock), and directed onto the interferometer coupled to the MPD.

### Parallel Rapid Imaging with Spectroscopic Mapping (PRISM)

PRISM is a rapid wide-field pump–probe imaging technique that enables direct mapping of exciton and carrier dynamics with acquisition times down to 50 ms. Ultrafast pump and probe pulses (∼32 fs duration) were generated from two independent second-harmonic oscillators, both pumped by a 1030 nm laser (Light Conversion, Carbide). The beams were weakly focused to a spot size of ∼80 μm^2^ at the sample plane to provide uniform wide-field illumination. The transmitted probe was collected with a 50×/0.42 NA objective (Mitutoyo, Plan Apo NIR) and the formed image was relayed to a high-speed camera (Phantom S710) operating at 20,000 frames per second while the pump–probe delay was continuously scanned. Delay control was achieved using a voice coil translation stage (Physik Instrumente, V-522) oscillating at 10 Hz with a retroreflector in the probe path. The delay corresponding to each frame was calibrated interferometrically using a copropagating HeNe laser, with the interference signal recorded by a photodetector (Thorlabs, PDA10A2) and digitized (AlazarTech, ATS9146). Camera frames were acquired through a frame grabber (Euresys, Coaxlink Octo), forming a three-dimensional data matrix where the first two axes corresponded to spatial coordinates and the third to pump–probe delay. All hardware components, including the laser, camera, digitizer, and frame grabber, were synchronized to ensure timing precision.

## Supplementary Material





## Notes

Additional growth details and experimental results (PDF)
Raw data tables for published results (XLSX)
