# Supplementary material for: Dynamic Behavior of Bound Interlayer Excitons in Interlayer-Doped Cs3Bi2Br9 Vacancy-Ordered Perovskite
Source: ACS Nano. 2025 Oct 16;19(42):37398–406. doi: 10.1021/acsnano.5c14651 (PMC12574210; doi:10.1021/acsnano.5c14651)
Supplement: Supplementary file 1 [file nn5c14651_si_001.pdf]

*Supporting Information for:*

# Dynamic Behavior of Bound Interlayer Excitons in Interlayer-Doped $Cs_3Bi_2Br_9$ Vacancy-Ordered Perovskite

Kyeongdeuk Moon,[1] Yang Ding,[2] Halyna Okrepka,[2] Rihan Wu,[1] Caitlin N. Ewald,[1] Pushpender Yadav,[1] Anupam Biswas,[1] Elad Harel,[1] Masaru Kuno,[2] Seokhyoung Kim[1]*

[1]*Department of Chemistry, Michigan State University, East Lansing, MI 48824, USA*

[2]*Department of Chemistry and Biochemistry, University of Notre Dame, Notre Dame, IN, 46556, USA*

**Supporting information includes:**

Figure S1: Schematic diagram of chemical vapor deposition (CVD) synthesis.

Figure S2: Experimental CVD growth parameters.

Figure S3: Optical images of CBB and Ag-CBB at low magnification.

Figure S4: Power-dependent PL.

Figure S5: TRPL of CBB at 480 nm with a single exponential fit.

Figure S6: TRPL of Ag-CBB at 680 nm with a biexponential fit.

Figure S7: Ultrafast transient absorption imaging of Ag-CBB and CBB.

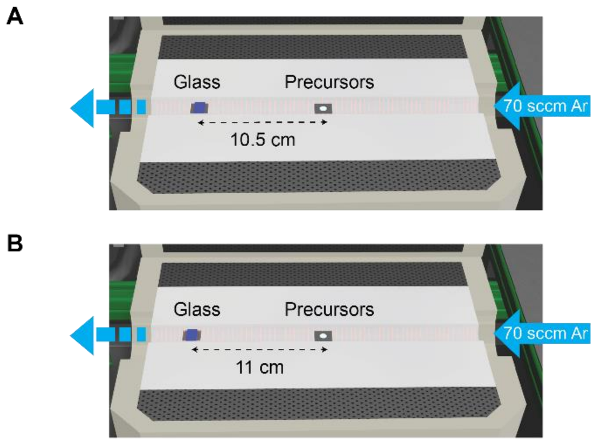


**Figure S1. Schematic diagram of chemical vapor deposition (CVD) synthesis.** (A) Ag doped $Cs_3Bi_2Br_9$ (Ag-CBB) particle growth. (B) $Cs_3Bi_2Br_9$ (CBB) particle growth. All precursors are mixed and placed in the center boat.

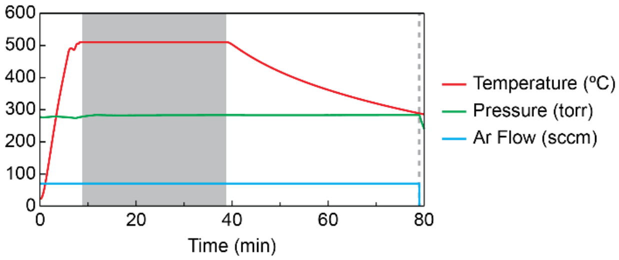


**Figure S2. Experimental CVD growth parameters.** Real-time recorded data of center temperature, reaction pressure, and argon gas flow rate. The grey area indicates the reaction period (30 minutes), and the grey dashed line marks the point at which argon flow is complete, and the quartz tube is ready to be pressurized to collect the substrate.

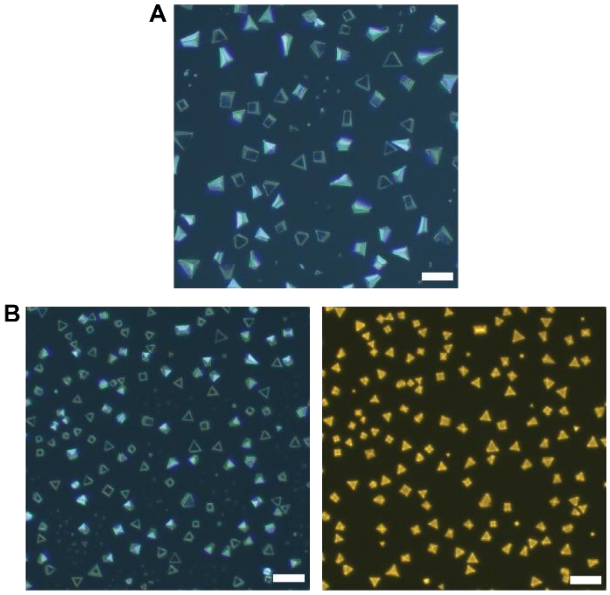


**Figure S3. Optical images of CBB and Ag-CBB at low magnification.** (A) Optical DF image of CVD-grown CBB. (B) Optical PL (left) and DF (right) images of CVD-grown Ag-CBB; scale bars are 20 µm.

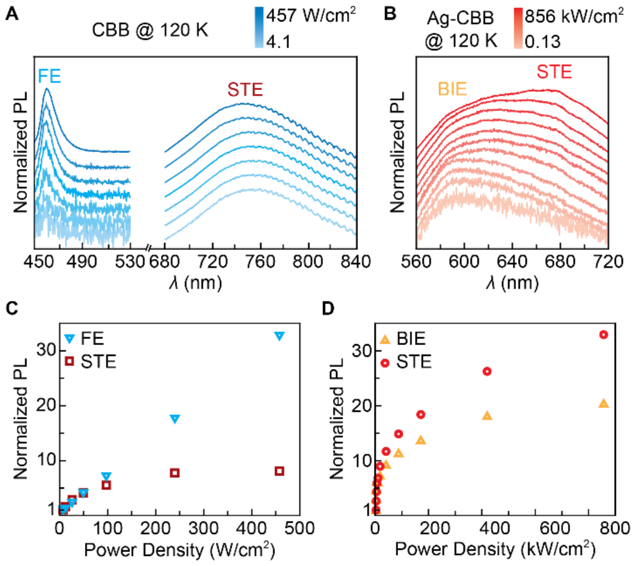


**Figure S4. Power-dependent PL.** (A-B) PL of CBB (A) and Ag-CBB (B) acquired from various excitation power density. (C-D) Normalized PL peak intensity as a function of excitation power density of CBB (C) and Ag-CBB (D).

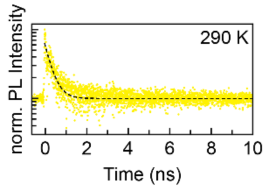


**Figure S5.** TRPL of CBB at 480 nm with a single exponential fit (black dashes).

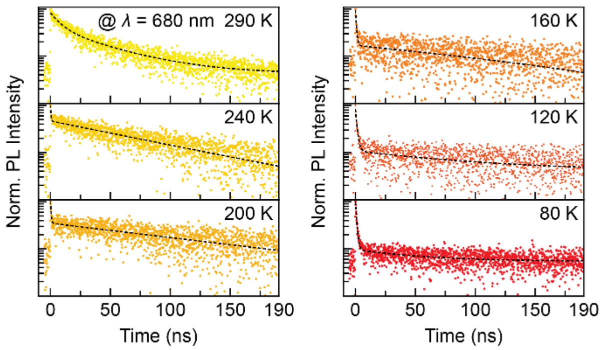


**Figure S6**. TRPL of Ag-CBB at 680 nm with bi-exponential fits (black dashes).

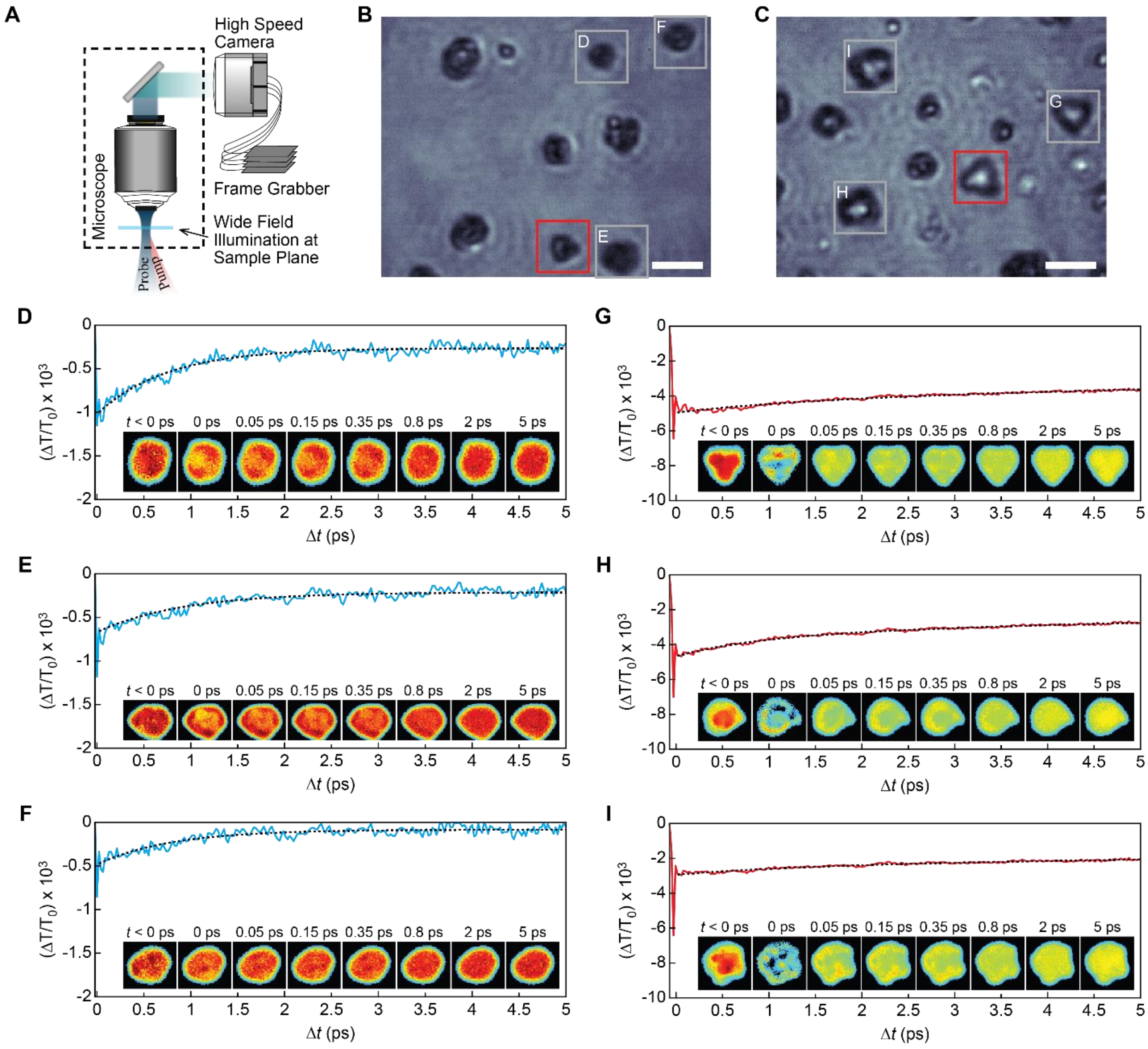


**Figure S7. Ultrafast transient absorption imaging of Ag-CBB and CBB.** (A) Scheme of transient absorption imaging. (B-C) Optical images of CBB (B) and Ag-CBB (C); scale bars, 20 μm. (D-I) Ultrafast transient absorption kinetics from single crystal within the grey box for CBB (D-F) and Ag-CBB (G-I), showing the normalized differential transmittance as a function of delay time. Inset images show percent transmittance change at various delay times.
